# Leveraging a cross-species probabilistic reward task (PRT) in suicide research. A commentary on Luc and Kangas (2024)

**DOI:** 10.3758/s13415-024-01181-0

**Published:** 2024-03-12

**Authors:** Steven J. Lamontagne, Carlos A. Zarate, Elizabeth D. Ballard

**Affiliations:** Experimental Therapeutics and Pathophysiology Branch, Intramural Research Program, National Institute of Mental Health, National Institutes of Health, Building 10, CRC Room 7-3331, 10 Center Drive, MSC 1282, Bethesda, MD 20892 USA

**Keywords:** Probabilistic reward task, Suicide, Animal models, Anhedonia, Ketamine

## Introduction

In this issue, Luc & Kangas (2023) describe the validation of a novel probabilistic reward task (PRT) designed for mice, outlining a rigorous and methodical approach that revealed cross-species continuity in task performance. Comparable task outcomes have been shown in other laboratory animal species (e.g., marmosets, rats) as well as humans, underscoring the remarkable translational value of the PRT. The authors correctly note the enormous potential for this task to inform a mechanistic understanding of anhedonia, as well as to address gaps in treatment options for reward learning dysfunction.

Our group has recently explored the development of objective laboratory-based tasks in suicide research and described the potential for animal models to inform suicide biomarkers and treatment efforts (Lamontagne et al., 2022). This much-needed endeavor has only begun to garner attention in the field, as a growing number of suicide researchers appreciate the need to complement self-reports with objective measures. This movement stems from recent articles describing the limitations associated with the subjective reporting of suicidal thoughts or behaviors (STBs), including concerns about patient misrepresentation or concealment of information, as well as issues surrounding the dynamic nature of suicidal thoughts.

Here, we comment on the broader utility of the PRT to advance our understanding of suicide risk and biological treatments to deter a crisis. Although STBs cannot be directly studied in nonhuman animals, risk factors clinically linked to STBs can be modeled and tested in a variety of species. By approaching suicide research from an endophenotype perspective (i.e., the study of underlying processes, like reward learning deficits, linked to suicide), studies can incorporate cross-species tasks to understand the mechanisms that contribute to suicide risk. In doing so, we encourage others to refine and develop translational tasks that will allow mechanistic insights into suicide, thereby promoting novel treatment targets for prevention.

## Nonhuman PRT in suicide biomarker research

The association between anhedonia and suicide remains complex. Although anhedonia has been reliably linked to suicidal ideation (SI), its relationship with suicidal behaviors remains inconclusive. This uncertainty is likely, in part, because of constraints associated with standard self-report measures of anhedonia. Indeed, some have argued that specific facets of anhedonia (e.g., consummatory vs. motivational) might be differentially related to SI versus behaviors, but these constructs are rarely parsed in self-report scales. While limited, some evidence points to deficits in task-based reward learning among suicide attempters (for a review and meta-analysis, see Sastre-Buades et al., 2021), suggesting that the inability to modify behavior according to differential reinforcement contingencies might be a precursor for suicidal behaviors.

Given its strong cross-species concordance, the PRT is ideally suited not only to clarify discrepancies in reward-related processes linked to STBs in humans but also could allow for mechanistic studies by using other species. Previous work in rats used pharmacological challenges and intracranial drug infusions to identify the neural circuitry underlying reward learning in the PRT. These included cortico-striatolimbic systems that also subserve emotion and impulse control. Indeed, human neuroimaging studies of individuals with STBs have identified connectivity abnormalities within similar reward networks (e.g., striatum, orbitofrontal cortex, thalamus) (for a comprehensive review of suicide neuroimaging studies, see Schmaal et al., 2020). These systems could therefore be manipulated in rodent models (e.g., perturbations caused by chronic stress or pharmacological challenge or ablation) to explore biomarkers of reward-related deficits in suicide.

Importantly, the mouse PRT described by Luc & Kangas (2023) presents a novel opportunity to study the genetic basis of suicide using gene knockout models. For example, suicide attempters showed reduced binding of the serotonin transporter (5-HTT) in the putamen (Schmaal et al., 2020). Genetic deletion of *5-HTT* in mice, which negatively impacts goal-directed reward learning, could become a critical tool to study biomarkers of treatment response in the context of suicide. Other polymorphisms linked to STBs could include dopamine pathway genes involved in synthesis, degradation, and receptor expression. Findings from these preclinical studies could then be applied directly to a clinical population using neuroimaging (e.g., functional magnetic resonance imaging or positron emission tomography).

## Exploring novel pharmacotherapies for suicide prevention

In addition to exploring biomarkers of suicide risk, the PRT could help to identify biomarkers of treatment response, which could ultimately accelerate drug development efforts for suicide prevention. As an interesting example, reward learning in the marmoset PRT was potentiated by intramuscular injections of ketamine—an N-methyl-D-aspartate (NMDA) receptor antagonist with rapid antidepressant and antisuicidal ideation properties (Wooldridge et al., 2021). These effects were not observed following administration of phencyclidine, a similar NMDA receptor antagonist with no known antidepressant effects. This suggests that ketamine’s mechanism of action may be uniquely linked to reward learning, which might include NMDA receptor-independent mechanisms, such as α-amino-3-hydroxy-5-methyl-4-isoxazolepropionic acid (AMPA) signaling.

Future cross-species efforts using the PRT could offer insight into the precise mechanisms of ketamine’s therapeutic actions, which remain poorly understood. To achieve this, studies might implement pharmacological challenges or genetic modifications in marmosets as previously discussed in rodents. Marmoset studies also could be used to explore the effects of ketamine on well-known, suicide-related risk factors that cannot be manipulated in human studies, such as early-life adversity or acute and chronic stress exposure. Examining these perturbations could elucidate causal mechanisms linked to suicide-related endophenotypes. Cross-validation with the human PRT could then allow for clinical applications, including the pursuit of novel rapid-acting agents that restore reward responsivity in at-risk populations. These efforts could help to determine how ketamine’s antisuicidal effects are mediated by its ability to normalize reward learning, offering mechanistic insights into its therapeutic action. It should be noted that although we have outlined a potential avenue to explore ketamine’s effects using the PRT, mechanistic animal studies could inform other targets that influence task performance, such as serotonin and dopamine. Such studies might shed light on the precise mechanisms implicated in current treatments for suicide: for example, the possibility that the ketamine metabolite hydroxynorketamine (HNK) modulates reward responsivity in the PRT. In turn, novel treatment options will emerge, and the PRT can directly test treatment efficacy in interventional clinical research due to its established cross-species concordance.

## Conclusions

In this special issue, Luc & Kangas (2023) describe exciting new directions for translational research in anhedonia. We believe that the PRT can serve as a prototype for developing novel cross-species tasks across many domains of human functioning. Furthermore, various traits linked to suicide have been successfully modeled in nonhuman species, including impulsivity, helplessness or despair, sleep disturbances, and aggression. PRT performance, as well as its neural underpinnings, could be evaluated in the context of these traits, thereby informing mechanisms associated with suicide-related risk factors. Likewise, candidate endophenotypes for suicidal behavior could be studied using specific challenges that cannot be conducted in human studies, such as drug administration or stress induction. Given the well-established convergence of task performance from rodents to primates, the translational potential of this research is remarkable. It offers the opportunity to deepen our understanding of suicide risk and, ultimately, develop more effective treatments to deter a crisis.

## Data Availability

Not applicable
